# Association between Bone Quality and Physical Activity in Community-Dwelling Older Adults

**DOI:** 10.3390/geriatrics9030062

**Published:** 2024-05-11

**Authors:** Koki Akai, Koutatsu Nagai, Shotaro Tsuji, Katsuyoshi Hirose, Daisuke Maruyama, Ryota Matsuzawa, Kayoko Tamaki, Hiroshi Kusunoki, Yosuke Wada, Ken Shinmura

**Affiliations:** 1 Department of Rehabilitation, JCHO Osaka Hospital, Osaka 553-0003, Japan; kohki.soccer14@gmail.com; 2 Department of Physical therapy, School of Rehabilitation, Hyogo Medical University, Kobe 650-8530, Japan; ri-matsuzawa@hyo-med.ac.jp; 3 Department of Orthopedic Surgery, Hyogo Medical University, Nishinomiya 663-8501, Japan; tj13041305sho@gmail.com; 4 Department of Rehabilitation, Matsushita Memorial Hospital, Osaka 570-8540, Japan; smilehappy14kh@gmail.com; 5 Department of Rehabilitation, Shimada Hospital, Osaka 583-0875, Japan; daimaru12m25d@gmail.com; 6 Department of General Internal Medicine, School of Medicine, Hyogo Medical University, Nishinomiya 663-8501, Japan; kayoko_tamaki@hotmail.com (K.T.); kusunoki1019@yahoo.co.jp (H.K.); pydss522@yahoo.co.jp (Y.W.); shimmura@hotmail.co.jp (K.S.); 7 Department of Internal Medicine, Osaka Dental University, Hirakata 573-1121, Japan; 8 Roppou Clinic, Toyooka 668-0851, Japan; 9 Department of General Medicine and Community Health Science, Sasayama Medical Center, Hyogo Medical University, Tambasasayama 669-2321, Japan

**Keywords:** bone quality, physical activity, bone mineral density

## Abstract

Bone quality is an essential factor determining bone strength. However, the relationship between physical activity (PA) and bone quality remains unclear. This study aimed to ascertain the relationship between bone quality and PA using a cortical bone quantitative ultrasound device that measures components of bone quality. In this cross-sectional study, bone quality was assessed in community-dwelling older adults by measuring the cortical speed of sound (cSOS) at the mid-tibia using a quantitative ultrasound device. Using a wrist-worn accelerometer, we calculated the daily duration of moderate-to-vigorous physical activity (MVPA) and light physical activity (LPA) based on estimated METs from the accelerometer data, without differentiating between types of activities. A multiple regression analysis was performed to examine the association between PA and the cSOS. The participants’ physical activity averaged 42.0 min/day for MVPA and 483.6 min/day for LPA. No significant association was observed between PA and bone quality in either men or women in the crude models. Furthermore, PA was not significantly correlated with the cSOS in the models adjusted for age, body mass index, nutrient intake, number of medications, and kidney disease. This study was a cross-sectional study which focused on the association between bone quality in older adults and their current amount of PA. The cSOS, as a measure of bone quality, was not associated with PA in men or women. Higher amounts of daily PA, as estimated from metabolic equivalents with an accelerometer, may not necessarily maintain or improve bone quality in older adults. This study does not rule out the potential for a positive association between PA levels or types and bone quality in younger or middle-aged individuals. It was specifically targeted at older adults, and its findings should not be generalized to younger populations. Further longitudinal studies are required to better understand the relationship between PA and bone quality.

## 1. Introduction

In recent years, the prevalence of skeletal disorders, including bone fractures and osteoporosis, among older adults has increased with the demographic trend towards an aging population. These pathologies exacerbate the global demand for nursing care and medical expenses [1]. Therefore, the prevention of skeletal diseases, particularly bone fractures and osteoporosis, among healthy older adults in the community is imperative.

Bone strength is acknowledged as a determinant of osteoporosis and fractures; the National Institute of Health states that bone strength “comprises two components, bone mineral density (BMD) and bone quality, with BMD explaining almost 70% of bone strength and bone quality accounting for the remaining 30%” [2]. Bone quality is defined by material properties, such as the quality of the bone material and its structural properties (microstructure, mineralization, microfracture, and bone turnover), which are built from that material [2]. In recent years, the traditional focus on BMD as a crucial determinant of bone strength has been revised with a renewed focus on the role of other factors, particularly bone quality.

BMD has been demonstrated to be associated with various factors such as hormones [3,4], smoking [5,6], nutritional intake [7,8], and alcohol consumption [9,10]. In particular, guidelines and numerous articles have reported that physical activity (PA) and exercise interventions are positively associated with BMD [11,12,13]. However, bone quality remains poorly understood owing to the limited number of methods available for its clinical assessment. As a result, there have been only a few studies investigating factors related to bone quality, and in particular, no studies have examined the relationship between PA and bone quality, which has been reported to have an impact on BMD.

Therefore, the purpose of this study was to ascertain the relationship between bone quality and PA using a cortical bone quantitative ultrasound (QUS) device that measures the cortical speed of sound (cSOS), which reflects cortical bone microstructure and mineralization and is a component of bone quality. Bone quality is a distinct component from bone mineral density (BMD) [14], which has been extensively studied. Understanding bone quality provides new insights into bone strength. Additionally, investigating its association with physical activity may lead to the discovery of new intervention targets for preventing skeletal diseases, particularly bone fractures and osteoporosis. It is hypothesized that a high amount of physical activity is involved in the maintenance and enhancement of bone quality among community-dwelling older adults.

## 2. Materials and Methods

This cross-sectional study was designed to be a study conducted on frail elderly people in the Sasayama-Tamba area. Study populations comprising individuals aged ≥ 65 years were recruited among community-dwelling older adults in the Sasayama-Tamba area, a rural area in Hyogo Prefecture, Japan, between April 2018 and December 2019. Individuals who (1) had been diagnosed with osteoporosis; (2) had taken osteoporosis medications or vitamin D; or (3) had missing data were excluded. The participants were informed of the study’s objectives and methods and provided written informed consent. The data collected in this study were anonymized and masked for analysis. This study received approval from the Ethics Review Board (approval number: Rinhi 0342) and was conducted in accordance with the principles of the Declaration of Helsinki.

### 2.1. Participant Characteristics

We assessed the following characteristics: age, body mass index (BMI), comorbidities, number of prescribed medications, bone quality, BMD, walking speed, grip strength, nutritional intake, and PA.

### 2.2. Bone Assessment

#### 2.2.1. BMD

To assess trabecular BMD, the right calcaneus was used as the measurement site, and the speed of sound (SOS) was measured using a QUS device (CM200, FURUNO ELECTRIC, Nishinomiya, Japan) [15]. The measurement protocol consisted of wiping the lateral surface of the right calcaneus with alcohol cotton, applying ultrasound gel to the tip of the standoff, and placing the right foot on a footrest in a sitting position. The position of the footrest was adjusted according to foot size, and the SOS was measured by clamping the center of the right calcaneus with standoffs.

#### 2.2.2. Bone Quality

To assess bone quality, the cSOS was measured at the mid-tibia using an axial transmission method with a QUS device (FURUNO ELECTRIC; Nishinomiya, Japan). During the assessment, a measurement brace was attached to each leg. This brace allowed the probe to be placed quickly and reproducibly in the same area. The probe was then slid horizontally along the brace for the measurement [14].

Bone quality is generally defined by the microstructure and degree of calcification of the bone tissue, bone turnover, and microfractures [16]. A previous study showed that the cSOS was strongly correlated with cortical bone tissue mineral density (TMD) (Ct. TMD), measured by high-resolution peripheral quantitative computed tomography; however, the SOS was not [14]. Ct. TMD is the BMD of the cortical bone, excluding areas with porosity, and reflects the degree of calcification and microporosity of the cortical bone. Thus, the cSOS is considered an indicator of microstructure and hypocalcification, which is a part of the bone structure [14]. Hence, we used the cSOS as an indicator of bone quality.

### 2.3. Physical Activity (PA)

PA was continuously measured for 14 days using a wrist-worn accelerometer (Actiband; TDK Corporation, Tokyo, Japan) with a minimum data storage unit of 5 min. Moderate-to-vigorous physical activity (MVPA) and light-intensity physical activity (LPA) were measured. The exercise intensity was defined as MVPA (≥3.0 metabolic equivalents, hereinafter referred to as METs) and LPA (1.5–3.0 METs), respectively, in accordance with previous studies [17]. The participants were instructed to wear the accelerometer at all times, including during sleep, but were permitted to remove it during bathing, if necessary. The accelerometers were returned by mail. The participants were blinded to the PA data during the measurement period. Data with <600 min of effective wearing time during the day were excluded because of insufficient wearing time [18]. Data from participants who had been wearing the device for at least 4 days during the 14-day measurement period were included in the analysis. The measured activity data were processed using MATLAB (MathWorks, Tokyo, Japan). The reliability and validity of the device have been confirmed in a previous study [19].

### 2.4. Measurement of Nutrient Intake

Eating habits in the previous month were assessed using the Brief Self-Reported Dietary History Questionnaire (BDHQ) [20,21]. The BDHQ is a structured 10-page questionnaire used to inquire about the frequency of consumption of commonly consumed foods in Japan, general eating behaviors, and usual cooking methods. Energy and nutrient intakes per day were calculated using the BDHQ responses and the Japanese Food Standard Composition Table [22], along with an ad hoc computer algorithm. BDHQ calculations were performed for all study participants with fixed sex-differentiated portions. The validity of the BDHQ has been described in detail in previous studies [20,21]. As calcium and vitamins D and K are widely known to affect BMD, they were used as nutritional indicators in this study [16]. All participants completed the BDHQ independently and were assisted in completing all questionnaires by a trained dietitian or researcher.

### 2.5. Statistics

Spearman’s rank correlation coefficient was used to examine the correlation between the cSOS and SOS and age, BMI, and PA (LPA and MVPA) by sex. To determine the association of cSOS and SOS with PA, a multiple regression analysis was performed with cSOS and SOS as dependent variables and PA (LPA and MVPA) as an independent variable (crude model). Subsequently, the models were adjusted for age, BMI, nutritional intake (calcium, vitamin D, and vitamin K), number of medications, and kidney disease as covariates that may affect the cSOS, the SOS, and PA (adjusted model). All analyses were performed with IBM SPSS version 24 (IBM Japan Ltd., Tokyo, Japan). Statistical significance was set at *p* < 0.05.

## 3. Results

Of the 513 participants in this study, we excluded those who were diagnosed with osteoporosis (*n* = 31) or who had taken osteoporosis medications or vitamin D (*n* = 14). Individuals with missing data on the survey measures (cSOS, MVPA, and nutritional data assessed using the questionnaire) were also excluded (*n* = 24). Therefore, 452 individuals were included in the final analysis.

**Figure 1:**
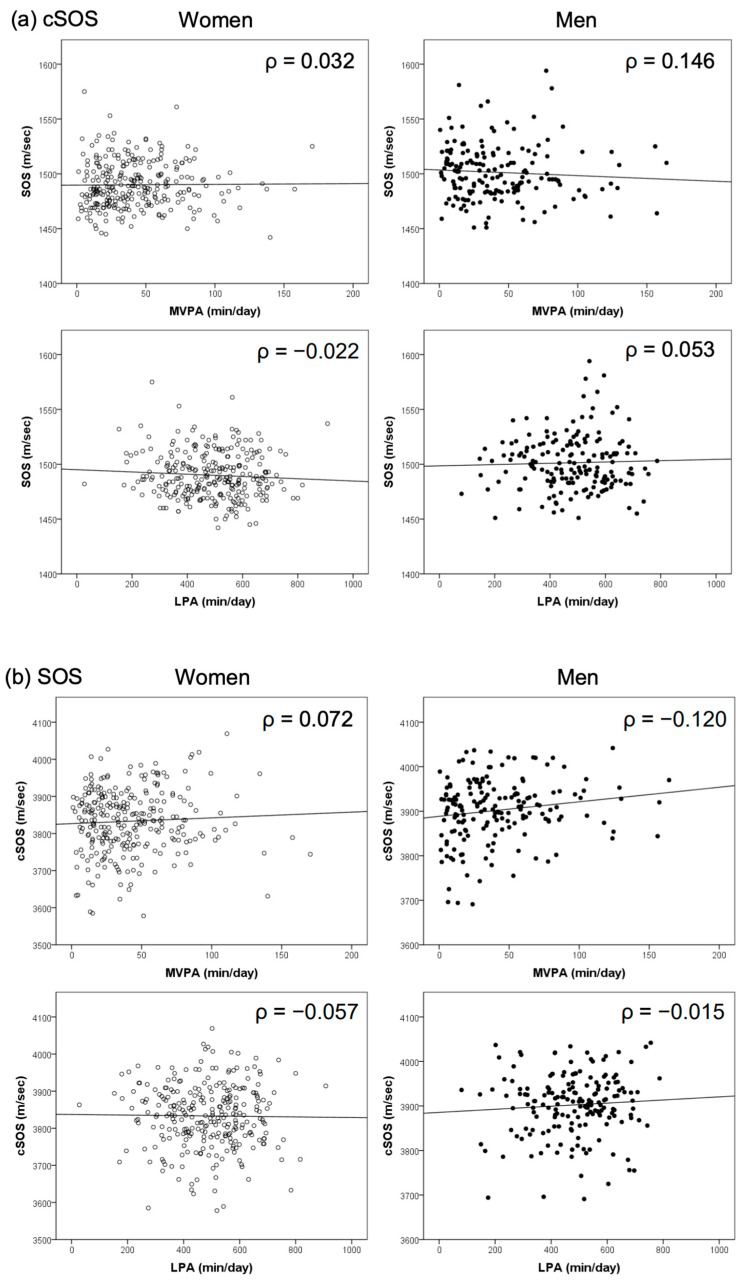
Correlation between cSOS and SOS and age, BMI, and physical activity (LPA, MVPA) by sex. Black circles represent men, and white circles represent women. ρ: Spearman’s rank correlation coefficient; cSOS: cortical speed of sound; SOS: speed of ultrasound; BMI: body mass index; MVPA: moderate-to-vigorous physical activity; LPA: light-intensity physical activity.

Among the basic attributes, the cSOS, the SOS, and grip strength were significantly different between men and women (all *p* < 0.001) (Table 1). There was no significant correlation between the cSOS, the SOS, and PA, including LPA and MVPA, in either sex (Table 2 and Figure 1). In the crude regression model, the cSOS was not significantly associated with PA (LPA or MVPA) in either men or women. The crude SOS model also revealed no significant associations. The models adjusted for age, BMI, nutrient intake (calcium, vitamin D, and vitamin K), number of medications, and kidney disease also showed no significant association with the cSOS, the SOS, or PA (Table 3).

## 4. Discussion

This study investigated the relationship between bone quality, a component of bone strength, and PA. The results indicated that the cSOS, which represents bone quality, was not significantly correlated with LPA or MVPA in either men or women. This finding suggests that bone quality may not be associated with PA in community-dwelling older adults, which implies that higher PA levels may not contribute to the maintenance or improvement of bone quality. Additionally, a supplementary analysis aimed at confirming the relationship between BMD and PA showed no significant association, indicating that PA may not have a significant impact on BMD in community-dwelling older adults.

In the univariate analysis, the cSOS, representing bone quality, showed no clear association with PA in either men or women. This was also the case for the SOS, which represents bone mineral density. Furthermore, in the multivariate analysis, which considered confounding factors, no significant association was found in either men or women. These results indicate that the independent and significant relationship between bone quality and PA is not remarkable, which leads to the suggestion of three possibilities that will be discussed later.

First, daily PA measured using an accelerometer may not be associated with bone quality. Meta-analyses examining the effects of exercise on maintaining or improving BMD have reported that exercise interventions increase BMD in the proximal femur and lumbar spine [11,12]; the effect of increased BMD was found in both cortical and trabecular bone [23]. Exercise is an important factor in maintaining BMD and affects both cortical and trabecular bones. These effects of exercise interventions are provided by combined exercises, such as resistance and loading exercises [12,24,25,26], and are greater with high-intensity exercises than with low- and moderate-intensity exercises [26,27]. This suggests that resistance or loading exercise should be of higher intensity and provide sufficient mechanical stimulation to the bones. However, this study measured daily PA using an accelerometer and was unable to measure the degree of weight-bearing load on the bones or the amount of resistance and loading exercises. Our findings suggest that the intensity and type of exercise may be important for increasing BMD and bone quality through PA. As in previous studies, higher-intensity exercises, such as resistance or load-bearing exercises, may increase bone quality and BMD. To better define the effects of PA on bone quality and BMD, the amounts of resistance and loading exercises should be quantified.

Second, current PA may not be an important factor for BMD or bone quality in older patients. To achieve strong bones in old age, it is essential to attain a high maximum BMD during early adulthood, particularly in women, to prevent rapid bone loss after menopause [16]. Studies examining the effects of PA in adolescence on BMD in adulthood have reported that higher levels of PA from adolescence [28] to young adulthood [29,30] are associated with the attainment of higher adult BMD and that leisure-time PA in adulthood after the attainment of high maximal BMD prevents the loss of BMD in early old age [31]. However, this study only measured current PA in old age and did not assess PA from the past to the present. Given the results of previous studies and the present study, the impact of PA on BMD and bone quality may be due to the cumulative weight-bearing load on the bones. Further research, especially longitudinal studies, is needed to better understand the effects of PA on bone quality.

Third, it was suggested that PA may not be linked to “structure,” a component of bone quality. Bone quality is defined by the properties of the bone material and its structural properties, such as microstructure, mineralization, microfracture, and bone turnover [2]. The cSOS in this study reflects porosity and mineralization, which are structural properties of bone quality, and not material properties [14]. Considering these results, the effect of PA on the “microstructure” may be limited in community-dwelling older adults.

Regarding gender, it was initially hypothesized that differences might exist in the association between physical activity (PA) and bone quality, given that hormones affecting bone metabolism vary between genders [32]. However, those results were negative. The present study was unable to confirm any gender differences in the relationship between PA and bone quality.

This study has several research limitations. First, it was a cross-sectional study that examined the association between PA and bone quality in older adults, not whether PA (amount or type) in younger or middle-aged individuals is associated with bone quality. Moreover, it was unable to determine the effect of the current amount of physical activity on bone quality in older adults. Longitudinal studies may shed light on the relationship between PA level and bone quality. Second, the study population was limited to a specific local population with the ability and willingness to attend the measurement sites independently. This raises the possibility of a selection bias and limits the generalizability of the results. Additionally, the participant population consisted of relatively active older adults who were either healthy or generally able to perform activities in daily living. Therefore, it is important to note that this research focused on older adults and does not deny the possibility of a positive association between PA and bone quality in younger or middle-aged individuals.

The third limitation involves measuring physical activity. This study assessed only the duration of physical activity, classifying it into two intensity categories, LPA and MVPA, based on METs inferred from the acceleration data. Detailed evaluations of higher intensities, such as VPA, or the nature of physical activity, including mechanical stimulations, were not conducted. Consequently, the analysis of how the nature of physical activity affects bone quality is limited, and the results regarding the relationship between activity duration and bone quality are inconclusive. 

Fourth, the study’s focus on tibial bone quality, which differs from regions where fractures commonly occur in older adults, introduces limitations. Thus, this study is limited in its reference to the clinical significance of the results as a direct contribution to the prevention of bone disease in older adults. Research on the variation in the cSOS in response to changes in bone quality is currently limited. It is necessary to conduct intervention studies to ascertain whether the cSOS possesses adequate sensitivity to detect all variations in bone quality.

This study is novel in its focus on factors related to bone quality, which differs from bone mineral density (BMD), a well-known determinant of bone strength. Despite growing interest, bone quality remains underexplored and poorly understood, making this study a meaningful contribution to bone health research. For future research, it is crucial to examine how bone quality is influenced by the nature of physical activity, the measurement site, and gender differences. 

## 5. Conclusions

This study investigated the relationship between bone quality and PA in older adults. The cSOS, which represents bone quality, was not correlated with PA. These results suggest that increasing daily PA does not necessarily lead to the maintenance or improvement of bone quality in older adults. Therefore, further longitudinal studies are required to elucidate the relationship between bone quality and PA.

## Figures and Tables

**Table 1:** Characteristics of participants.

Variables ^1^	Overall (*n* = 452)	Women (*n* = 278)	Men (*n* = 174)	*p*-Value
Age, years, mean (SD)	72.2 (5.8)	71.8 (5.5)	73.0 (6.1)	0.030
BMI, kg/m^2^, mean (SD)	22.6 (2.9)	22.2 (2.9)	23.0 (2.8)	0.004
Diabetes, n (%)	64 (14.1)	30 (10.8)	34 (19.5)	0.009
Kidney disease, n (%)	13 (2.9)	5 (1.8)	8 (4.6)	0.117
Number of drugs taken, mean (SD)	1.6 (1.9)	1.4 (1.6)	1.9 (2.2)	0.008
tib cSOS, m/s, mean (SD)	3859.8 (86.8)	3833.1 (85.9)	3902.3 (69.8)	<0.001
SOS, m/s, mean (SD)	1494.4 (23.3)	1490.0 (21.2)	1501.3 (24.9)	<0.001
Nutritional intake				
Calcium (Ca), mg/day, mean (SD)	790.4 (330.7)	809.5 (299.3)	759.8 (374.4)	0.140
Vitamin D, μg/day, mean (SD)	23.8 (16.5)	24.8 (16.3)	22.2 (16.9)	0.106
Vitamin K, μg/day, mean (SD)	412.6 (216.9)	420.7 (202.6)	399.7 (238.1)	0.319
Physical activity				
MVPA, min/day, mean (SD)	42.0 (31.3)	42.0 (29.5)	42.1 (34.0)	0.960
LPA, min/day, mean (SD)	483.6 (139.5)	486.2 (139.3)	479.4 (140.1)	0.614
**Variables**	**Overall (*n* = 449)**	**Women (*n* = 277)**	**Men (*n* = 172)**	***p*-Value**
Gait speed, m/s, mean (SD)	1.5 (0.2)	1.5 (0.2)	1.5 (0.2)	0.372
Grip strength, kg, mean (SD)	28.7 (7.3)	24.6 (4.5)	35.3 (6.0)	<0.001

^1^ SD: standard deviation; BMI: body mass index; cSOS: cortical speed of sound; SOS: speed of ultrasound; MVPA: moderate-to-vigorous physical activity; LPA: light-intensity physical activity.

**Table 2:** Correlation between cSOS and SOS and age, BMI, and physical activity (LPA, MVPA) by sex.

(A) Women				
			PA	
	Age	BMI	MVPA	LPA
Cortical QUS (mid-tibia) cSOS	−0.189 **	0.063	0.032	−0.022
Calcaneus QUS SOS	−0.151 *	0.088	0.072	−0.057
(B) Men				
	Age	BMI	MVPA	LPA
Cortical QUS (mid-tibia) cSOS	−0.111	−0.023	0.146	0.053
Calcaneus QUS SOS	−0.147	0.224 **	−0.120	−0.015

cSOS: cortical speed of sound; SOS: speed of ultrasound; BMI: body mass index; PA: physical activity; MVPA: moderate-to-vigorous physical activity; LPA: light-intensity physical activity. Spearman’s rank correlation coefficient; * *p* < 0.05; ** *p* < 0.01.

**Table 3:** Results of multiple regression analysis.

Models	Subjects	B	β-Coefficient	SE	*p*-Value	R^2^
cSOS						
MVPA						
Crude model	Women	0.17	0.058	0.179	0.344	0.003
	Men	0.31	0.151	0.16	0.054	0.026
Adjusted model	Women	0.195	0.067	0.176	0.267	0.091
	Men	0.247	0.133	0.161	0.091	0.091
LPA						
Crude model	Women	−0.016	−0.025	0.038	0.681	0.003
	Men	0.016	0.032	0.039	0.677	0.026
Adjusted model	Women	−0.023	−0.038	0.038	0.536	0.091
	Men	−0.01	−0.02	0.04	0.807	0.091
SOS						
MVPA						
Crude model	Women	0.017	0.024	0.044	0.692	0.005
	Men	−0.061	−0.084	0.057	0.288	0.008
Adjusted model	Women	0.024	0.033	0.044	0.592	0.062
	Men	−0.07	−0.096	0.056	0.215	0.122
LPA						
Crude model	Women	−0.011	−0.073	0.009	0.237	0.005
	Men	0.01	0.054	0.014	0.492	0.008
Adjusted model	Women	−0.011	−0.074	0.009	0.233	0.062
	Men	0.011	0.063	0.014	0.426	0.122

Crude model: not adjusted for any variable; adjusted model: adjusted for age, body mass index, nutritional intake (calcium, vitamin D, and vitamin K), number of drugs taken, and kidney disease. cSOS: cortical speed of sound; SOS: speed of ultrasound; MVPA: moderate-to-vigorous physical activity; LPA: light-intensity physical activity.

## Data Availability

The data are available from the corresponding author, K.N., upon reasonable request.
